# Supplementary material for: Toward surface orbitronics: giant orbital magnetism from the orbital Rashba effect at the surface of sp-metals
Source: Sci Rep. 2017 Apr 25;7:46742. doi: 10.1038/srep46742 (PMC5404270; doi:10.1038/srep46742)
Supplement: Supplementary Information [file srep46742-s1.pdf]

Supplementary Information for "Toward surface orbitronics: giant orbital magnetism from the orbital Rashba effect at the surface of sp-metals"

Dongwook Go, Jan-Philipp Hanke, Patrick M. Buhl, Frank Freimuth,
Gustav Bihlmayer, Hyun-Woo Lee, Yuriy Mokrousov, and Stefan Blügel

Supplementary Note 1: Definition of the tight-binding model

Here, we present a tight-binding model for BiAg_2. The Bravais lattice vectors are defined as

$$\mathbf{R} = n_1\mathbf{a}_1 + n_2\mathbf{a}_2, \tag{S1}$$

where n_1 and n_2 are integers and

$$\mathbf{a}_1 = a\left(\frac{1}{2}\hat{\mathbf{x}} + \frac{\sqrt{3}}{2}\hat{\mathbf{y}}\right), \tag{S2}$$

$$\mathbf{a}_2 = a\left(\frac{1}{2}\hat{\mathbf{x}} - \frac{\sqrt{3}}{2}\hat{\mathbf{y}}\right), \tag{S3}$$

are unit lattice vectors, where a is the lattice constant. For convenience, we also define $\mathbf{a}_3 = -\left(\mathbf{a}_1 + \mathbf{a}_2\right)$. Within each unit cell, there are one Bi and two Ag atoms, the locations of which are

$$\mathbf{r}_{\mathrm{Bi}} = 0, \tag{S4}$$

$$\mathbf{r}_{\mathrm{Ag1}} = \frac{2}{3}\mathbf{a}_1 + \frac{1}{3}\mathbf{a}_2, \tag{S5}$$

$$\mathbf{r}_{\mathrm{Ag2}} = -\mathbf{r}_{\mathrm{Ag1}}. \tag{S6}$$

We first consider a spinless model. We will return to a spin-full model when we consider the effect of spin-orbit coupling. Near the Fermi energy, the most relevant atomic orbitals are the p_x, p_y, p_z orbitals of Bi atoms, and s orbitals of Ag atoms, which are sufficient to capture the essential features of the orbital Rashba effect in BiAg_2. We define $\phi_{p_\alpha}(\mathbf{r})$ $(\alpha = x, y, z)$ and $\phi_s(\mathbf{r})$ as p_α- and s-like Wannier functions localized around $\mathbf{r} = 0$, respectively. Considering up to the first- and second-nearest hoppings, we define the following hopping amplitudes:

$$E_s = +\int d\mathbf{r}\phi_s^*(\mathbf{r})\hat{H}\phi_s(\mathbf{r}), \tag{S7}$$

$$E_p = +\int d\mathbf{r}\phi_{p_x}^*(\mathbf{r})\hat{H}\phi_{p_x}(\mathbf{r}) = +\int d\mathbf{r}\phi_{p_y}^*(\mathbf{r})\hat{H}\phi_{p_y}(\mathbf{r}) = +\int d\mathbf{r}\phi_{p_z}^*(\mathbf{r})\hat{H}\phi_{p_z}(\mathbf{r}), \tag{S8}$$

$$t_{p\sigma} = +\int d\mathbf{r}\phi_{p_x}^*(\mathbf{r})\hat{H}\phi_{p_x}(\mathbf{r}\pm a\hat{\mathbf{x}}) = +\int d\mathbf{r}\phi_{p_y}^*(\mathbf{r})\hat{H}\phi_{p_y}(\mathbf{r}\pm a\hat{\mathbf{y}}), \tag{S9}$$

$$\begin{aligned} t_{p\pi} &= -\int d\mathbf{r}\phi_{p_x}^*(\mathbf{r})\hat{H}\phi_{p_x}(\mathbf{r}\pm a\hat{\mathbf{y}}) = -\int d\mathbf{r}\phi_{p_y}^*(\mathbf{r})\hat{H}\phi_{p_y}(\mathbf{r}\pm a\hat{\mathbf{x}}) \\ &= -\int d\mathbf{r}\phi_{p_z}^*(\mathbf{r})\hat{H}\phi_{p_z}(\mathbf{r}\pm a\hat{\mathbf{x}}) = -\int d\mathbf{r}\phi_{p_z}^*(\mathbf{r})\hat{H}\phi_{p_z}(\mathbf{r}\pm a\hat{\mathbf{y}}), \end{aligned} \tag{S10}$$

$$t_{s(1)} = -\int d\mathbf{r}\phi_s^*(\mathbf{r})\hat{H}\phi_s(\mathbf{r}\pm\frac{a}{\sqrt{3}}\hat{\mathbf{x}}) = -\int d\mathbf{r}\phi_s^*(\mathbf{r})\hat{H}\phi_s(\mathbf{r}\pm\frac{a}{\sqrt{3}}\hat{\mathbf{y}}), \tag{S11}$$

$$t_{s(2)} = -\int d\mathbf{r}\phi_s^*(\mathbf{r})\hat{H}\phi_s(\mathbf{r}\pm a\hat{\mathbf{x}}) = -\int d\mathbf{r}\phi_s^*(\mathbf{r})\hat{H}\phi_s(\mathbf{r}\pm a\hat{\mathbf{y}}), \tag{S12}$$

$$\gamma_{sp} = \pm\int d\mathbf{r}\phi_s^*(\mathbf{r})\hat{H}\phi_{p_x}(\mathbf{r}\mp\frac{a}{\sqrt{3}}\hat{\mathbf{x}}) = \pm\int d\mathbf{r}\phi_s^*(\mathbf{r})\hat{H}\phi_{p_y}(\mathbf{r}\mp\frac{a}{\sqrt{3}}\hat{\mathbf{y}}), \tag{S13}$$

$$V_{z(1)} = -\int d\mathbf{r}\phi_s^*(\mathbf{r})\hat{H}\phi_{p_z}(\mathbf{r}\pm\frac{a}{\sqrt{3}}\hat{\mathbf{x}}) = -\int d\mathbf{r}\phi_s^*(\mathbf{r})\hat{H}\phi_{p_z}(\mathbf{r}\pm\frac{a}{\sqrt{3}}\hat{\mathbf{y}}), \tag{S14}$$

$$V_{z(2)} = \pm\int d\mathbf{r}\phi_{p_x}^*(\mathbf{r})\hat{H}\phi_{p_z}(\mathbf{r}\pm a\hat{\mathbf{x}}) = \pm\int d\mathbf{r}\phi_{p_y}^*(\mathbf{r})\hat{H}\phi_{p_z}(\mathbf{r}\pm a\hat{\mathbf{y}}). \tag{S15}$$

Note that $V_{z(1)}$ and $V_{z(2)}$ are zero if z-inversion symmetry is preserved. The effect of $V_{z(2)}$ was studied by Petersen *et al.* [S1]. In sp alloys such as BiAg_2, the quantity $V_{z(1)}$ arising due to the buckling of Bi atoms becomes important.

In order to find a $\mathbf{k}$-space representation of the Hamiltonian, let us define basis states

$$\varphi_{n\mathbf{k}}(\mathbf{r}) = \frac{1}{\sqrt{N}} \sum_{\mathbf{R}} e^{i\mathbf{k}\cdot\mathbf{R}} \phi_n(\mathbf{r} - \mathbf{r}_n - \mathbf{R}), \tag{S16}$$

where $n = p_x,\ p_y,\ p_z,\ s_1,\ s_2$, and $\mathbf{r}_{s_{1(2)}} = \mathbf{r}_{\text{Ag1(2)}}$, and N is the number of lattice sites. We compute a matrix representation of the Hamiltonian as

$$H_{nm}(\mathbf{k}) = \int d\mathbf{r} \varphi^*_{n\mathbf{k}}(\mathbf{r}) \hat{H} \varphi_{m\mathbf{k}}(\mathbf{r}), \tag{S17}$$

where

$$H_{p_xp_x}(\mathbf{k}) = \left(\frac{1}{2}t_{p\sigma} - \frac{3}{2}t_{p\pi}\right)\cos(\mathbf{k}\cdot\mathbf{a}_1) + \left(\frac{1}{2}t_{p\sigma} - \frac{3}{2}t_{p\pi}\right)\cos(\mathbf{k}\cdot\mathbf{a}_2) + 2t_{p\sigma}\cos(\mathbf{k}\cdot\mathbf{a}_3), \tag{S18}$$

$$H_{p_yp_x}(\mathbf{k}) = \left(\frac{\sqrt{3}}{2}t_{p\sigma} + \frac{1}{2}t_{p\pi}\right)\cos(\mathbf{k}\cdot\mathbf{a}_1) - \left(\frac{\sqrt{3}}{2}t_{p\sigma} + \frac{1}{2}t_{p\pi}\right)\cos(\mathbf{k}\cdot\mathbf{a}_2), \tag{S19}$$

$$H_{p_yp_y}(\mathbf{k}) = \left(\frac{3}{2}t_{p\sigma} - \frac{1}{2}t_{p\pi}\right)\cos(\mathbf{k}\cdot\mathbf{a}_1) + \left(\frac{3}{2}t_{p\sigma} - \frac{1}{2}t_{p\pi}\right)\cos(\mathbf{k}\cdot\mathbf{a}_2) - 2t_{p\pi}\cos(\mathbf{k}\cdot\mathbf{a}_3), \tag{S20}$$

$$H_{p_zp_x}(\mathbf{k}) = -iV_{z(2)}\sin(\mathbf{k}\cdot\mathbf{a}_1) - iV_{z(2)}\sin(\mathbf{k}\cdot\mathbf{a}_2) + 2iV_{z(2)}\sin(\mathbf{k}\cdot\mathbf{a}_3), \tag{S21}$$

$$H_{p_zp_y}(\mathbf{k}) = -\sqrt{3}iV_{z(2)}\sin(\mathbf{k}\cdot\mathbf{a}_1) + \sqrt{3}iV_{z(2)}\sin(\mathbf{k}\cdot\mathbf{a}_2), \tag{S22}$$

$$H_{p_zp_z}(\mathbf{k}) = -2t_{p\pi}\cos(\mathbf{k}\cdot\mathbf{a}_1) - 2t_{p\pi}\cos(\mathbf{k}\cdot\mathbf{a}_2) - 2t_{p\pi}\cos(\mathbf{k}\cdot\mathbf{a}_3), \tag{S23}$$

$$H_{s_1s_1}(\mathbf{k}) = -2t_{s2}\cos(\mathbf{k}\cdot\mathbf{a}_1) - 2t_{s2}\cos(\mathbf{k}\cdot\mathbf{a}_2) - 2t_{s2}\cos(\mathbf{k}\cdot\mathbf{a}_3), \tag{S24}$$

$$H_{s_2s_1}(\mathbf{k}) = -t_{s1}e^{-i\mathbf{k}\cdot\mathbf{a}_1} - t_{s1}e^{i\mathbf{k}\cdot\mathbf{a}_3} - t_{s1}e^{i\mathbf{k}\cdot(\mathbf{a}_3-\mathbf{a}_1)}, \tag{S25}$$

$$H_{s_2s_2}(\mathbf{k}) = -2t_{s2}\cos(\mathbf{k}\cdot\mathbf{a}_1) - 2t_{s2}\cos(\mathbf{k}\cdot\mathbf{a}_2) - 2t_{s2}\cos(\mathbf{k}\cdot\mathbf{a}_3), \tag{S26}$$

$$H_{s_1p_x}(\mathbf{k}) = -\frac{\sqrt{3}}{2}t_{sp} + \frac{\sqrt{3}}{2}t_{sp}e^{-i\mathbf{k}\cdot\mathbf{a}_3}, \tag{S27}$$

$$H_{s_1p_y}(\mathbf{k}) = -\frac{1}{2}\gamma_{sp} + \gamma_{sp}e^{i\mathbf{k}\cdot\mathbf{a}_1} - \frac{1}{2}\gamma_{sp}e^{-i\mathbf{k}\cdot\mathbf{a}_3}, \tag{S28}$$

$$H_{s_1p_z}(\mathbf{k}) = V_{z(1)} + V_{z(1)}e^{i\mathbf{k}\cdot\mathbf{a}_1} + V_{z(1)}e^{-i\mathbf{k}\cdot\mathbf{a}_3}, \tag{S29}$$

$$H_{s_2p_x}(\mathbf{k}) = \frac{\sqrt{3}}{2}\gamma_{sp} - \frac{\sqrt{3}}{2}\gamma_{sp}e^{i\mathbf{k}\cdot\mathbf{a}_3}, \tag{S30}$$

$$H_{s_2p_y}(\mathbf{k}) = \frac{1}{2}\gamma_{sp} - \gamma_{sp}e^{-i\mathbf{k}\cdot\mathbf{a}_1} + \frac{1}{2}\gamma_{sp}e^{i\mathbf{k}\cdot\mathbf{a}_3}, \tag{S31}$$

$$H_{s_2p_z}(\mathbf{k}) = V_{z(1)} + V_{z(1)}e^{-i\mathbf{k}\cdot\mathbf{a}_1} + V_{z(1)}e^{i\mathbf{k}\cdot\mathbf{a}_3}. \tag{S32}$$

The above equations define a spin-less model Hamiltonian. A spin-full model Hamiltonian can be constructed by simply adding spin-orbit coupling as

$$H(\mathbf{k}) = \begin{pmatrix} H_{p_xp_x}\mathrm{I}_{2\times2} & H_{p_xp_y}\mathrm{I}_{2\times2} & H_{p_xp_z}\mathrm{I}_{2\times2} & H_{p_xs_1}\mathrm{I}_{2\times2} & H_{p_xs_2}\mathrm{I}_{2\times2} \\ H_{p_yp_x}\mathrm{I}_{2\times2} & H_{p_yp_y}\mathrm{I}_{2\times2} & H_{p_yp_z}\mathrm{I}_{2\times2} & H_{p_ys_1}\mathrm{I}_{2\times2} & H_{p_ys_2}\mathrm{I}_{2\times2} \\ H_{p_zp_x}\mathrm{I}_{2\times2} & H_{p_zp_y}\mathrm{I}_{2\times2} & H_{p_zp_z}\mathrm{I}_{2\times2} & H_{p_zs_1}\mathrm{I}_{2\times2} & H_{p_zs_2}\mathrm{I}_{2\times2} \\ H_{s_1p_x}\mathrm{I}_{2\times2} & H_{s_1p_y}\mathrm{I}_{2\times2} & H_{s_1p_z}\mathrm{I}_{2\times2} & H_{s_1s_1}\mathrm{I}_{2\times2} & H_{s_1s_2}\mathrm{I}_{2\times2} \\ H_{s_2p_x}\mathrm{I}_{2\times2} & H_{s_2p_y}\mathrm{I}_{2\times2} & H_{s_2p_z}\mathrm{I}_{2\times2} & H_{s_2s_1}\mathrm{I}_{2\times2} & H_{s_2s_2}\mathrm{I}_{2\times2} \end{pmatrix} + H_{\text{soc}}, \tag{S33}$$

where

$$H_{\text{soc}} = \frac{\lambda_{\text{soc}}}{2\hbar^2}\hat{\mathbf{L}}\cdot\hat{\mathbf{S}} = \lambda_{\text{soc}} \begin{pmatrix} 0 & 0 & -i & 0 & 0 & 1 & 0 & 0 & 0 & 0 \\ 0 & 0 & 0 & i & -1 & 0 & 0 & 0 & 0 & 0 \\ i & 0 & 0 & 0 & 0 & -i & 0 & 0 & 0 & 0 \\ 0 & -i & 0 & 0 & -i & 0 & 0 & 0 & 0 & 0 \\ 0 & -1 & 0 & i & 0 & 0 & 0 & 0 & 0 & 0 \\ 1 & 0 & i & 0 & 0 & 0 & 0 & 0 & 0 & 0 \\ 0 & 0 & 0 & 0 & 0 & 0 & 0 & 0 & 0 & 0 \\ 0 & 0 & 0 & 0 & 0 & 0 & 0 & 0 & 0 & 0 \\ 0 & 0 & 0 & 0 & 0 & 0 & 0 & 0 & 0 & 0 \\ 0 & 0 & 0 & 0 & 0 & 0 & 0 & 0 & 0 & 0 \end{pmatrix} \tag{S34}$$

is the spin-orbit coupling Hamiltonian at the intra-atomic level.

Supplementary Note 2: Parameters of the tight-binding model

The parameters occuring in the above tight-binding model were chosen as

$$E_s = 1,\ E_p = 0,\ t_{p\sigma} = 0.7,\ t_{p\pi} = 0,\ t_{s(1)} = 1.5,\ t_{s(2)} = 0,\ \gamma_{sp} = 1.3,\ V_{z(1)} = 0.6,\ V_{z(2)} = 0,\ \lambda_{soc} = 1.2,$$

in order to reproduce the band structure which resembles the result obtained from the first principles calculation (see Supplementary Figure 1). The Fermi energy is introduced as a parameter, and we set $E_F = 0.6$ and $E_F = 0.75$ for the case without and with spin-orbit coupling, respectively, such that the location of the spin-split bands near the Gamma-point is similar to that of the first-principles calculation. All parameters are given in electronvolt.

Supplementary Note 3: Derivation of Eq. (5)

In order to derive the orbital Rashba Hamiltonian from a tight-binding model of the sp-alloy, let us consider a two-dimensional square lattice in the xy-plane with three p orbitals and one s orbital at each site. A generalization to different lattice systems of sp-alloys is straightforward. The tight-binding Hamiltonian is written as

$$H(\mathbf{k}) = \begin{pmatrix} H_p(\mathbf{k}) & h(\mathbf{k}) \\ h^\dagger(\mathbf{k}) & H_s(\mathbf{k}) \end{pmatrix}, \tag{S35}$$

where the basis states are $|\varphi_{n\mathbf{k}}\rangle = (1/\sqrt{N}) \sum_{\mathbf{R}} e^{i\mathbf{k}\cdot\mathbf{R}} |\phi_{n\mathbf{R}}\rangle$ with $|\phi_{n\mathbf{R}}\rangle$ as the n-th $(n = p_x, p_y, p_z, s)$ Wannier function localized around the Bravais lattice vector $\mathbf{R}$, and N is the total number of the lattice sites. Here, $H_{p(s)}$ is the Hamiltonian spanned by $p(s)$ orbitals, i.e., $H_p(\mathbf{k}) = \text{diag}\left[E_{p_x}(\mathbf{k}), E_{p_y}(\mathbf{k}), E_{p_z}(\mathbf{k})\right]$, and $H_s(\mathbf{k}) = E_s(\mathbf{k})$, where $E_n(\mathbf{k})$ where $E_n(\mathbf{k})$ refers to the energy dispersion of the n-th isolated basis state. Most importantly,

$$h(\mathbf{k}) = \begin{pmatrix} i\gamma_{sp}\sin(k_x a) & i\gamma_{sp}\sin(k_y a) & V_z(\mathbf{k}) \end{pmatrix}^{\mathrm{T}} \tag{S36}$$

describes the hybridization between s and p orbitals, where γ_{sp} is the nearest-neighbor hopping amplitude between s and $p_{x(y)}$ orbitals, and $V_z(\mathbf{k})$ is the hopping amplitude between p_z and s orbitals, which is induced by the surface potential gradient. Near $\mathbf{k} = 0$,

$$h(\mathbf{k}) \approx \begin{pmatrix} i\gamma_{sp}k_x a & i\gamma_{sp}k_y a & V_z(0) \end{pmatrix}^{\mathrm{T}} \tag{S37}$$

up to the first order in $\mathbf{k}$. Considering $\mathbf{k}$ and $V_z(0)$ as perturbative parameters, the eigenstates of Eq. (S35) are given by

$$|\varphi'_{p_x\mathbf{k}}\rangle \approx |\varphi_{p_x\mathbf{k}}\rangle - \frac{i\gamma_{sp}k_x a}{E_{p_x}(0) - E_s(0)} |\varphi_{s\mathbf{k}}\rangle, \tag{S38}$$

$$|\varphi'_{p_y\mathbf{k}}\rangle \approx |\varphi_{p_y\mathbf{k}}\rangle - \frac{i\gamma_{sp}k_y a}{E_{p_y}(0) - E_s(0)} |\varphi_{s\mathbf{k}}\rangle, \tag{S39}$$

$$|\varphi'_{p_z\mathbf{k}}\rangle \approx |\varphi_{p_z\mathbf{k}}\rangle + \frac{V_z(0)}{E_{p_z}(0) - E_s(0)} |\varphi_{s\mathbf{k}}\rangle, \tag{S40}$$

$$|\varphi'_{s\mathbf{k}}\rangle \approx |\varphi_{s\mathbf{k}}\rangle + \frac{i\gamma_{sp}k_x a}{E_s(0) - E_{p_x}(0)} |\varphi_{p_x\mathbf{k}}\rangle + \frac{i\gamma_{sp}k_y a}{E_s(0) - E_{p_y}(0)} |\varphi_{p_y\mathbf{k}}\rangle + \frac{V_z(0)}{E_s(0) - E_{p_z}(0)} |\varphi_{p_z\mathbf{k}}\rangle. \tag{S41}$$

Using these basis states to obtain a matrix representation of the Hamiltonian Eq.(S35), we arrive at an effective Hamiltonian decoupling the manifolds of s and p orbitals:

$$H_{\text{eff}}(\mathbf{k}) = \begin{pmatrix} H_{p,\text{eff}}(\mathbf{k}) & 0 \\ 0 & H_{s,\text{eff}}(\mathbf{k}) \end{pmatrix}. \tag{S42}$$

In particular for the effective Hamiltonian describing the manifold of p orbitals [Eq. (S42)], we note the occurence of the so-called orbital Rashba Hamiltonian:

$$H_{p,\text{eff}}(\mathbf{k}) = H_p(\mathbf{k}) + H_{\text{OR}}(\mathbf{k}), \tag{S43}$$

where

$$H_{\text{OR}}(\mathbf{k}) = \frac{\alpha_{\text{OR}}}{\hbar} \hat{\mathbf{L}} \cdot (\hat{\mathbf{z}} \times \mathbf{k}). \tag{S44}$$

The orbital Rashba constant α_{OR} is given by

$$\alpha_{\text{OR}} = a\gamma_{sp}V_z(0)\left[\frac{1}{E_{p_{xy}}(0)-E_s(0)}+\frac{1}{E_{p_z}(0)-E_s(0)}\right], \tag{S45}$$

where we assumed $E_{p_x}(0) = E_{p_y}(0) \equiv E_{p_{xy}}(0)$. The orbital angular momentum operator in a given basis is defined as

$$\hat{L}_x = \hbar\begin{pmatrix}0 & 0 & 0\\ 0 & 0 & -i\\ 0 & i & 0\end{pmatrix},\ \hat{L}_y = \hbar\begin{pmatrix}0 & 0 & i\\ 0 & 0 & 0\\ -i & 0 & 0\end{pmatrix},\ \hat{L}_z = \hbar\begin{pmatrix}0 & -i & 0\\ i & 0 & 0\\ 0 & 0 & 0\end{pmatrix}, \tag{S46}$$

which satisfy the canonical commutation relations of the angular momentum, $[\hat{L}_\alpha, \hat{L}_\beta] = i\hbar\epsilon_{\alpha\beta\gamma}\hat{L}_\gamma$

[S1] L. Petersen and P. Hedegård, *A simple tight-binding model of spin-orbit splitting of sp-derived surface states*, Surf. Sci. **459**, 49 (2000).

Supplementary Figures

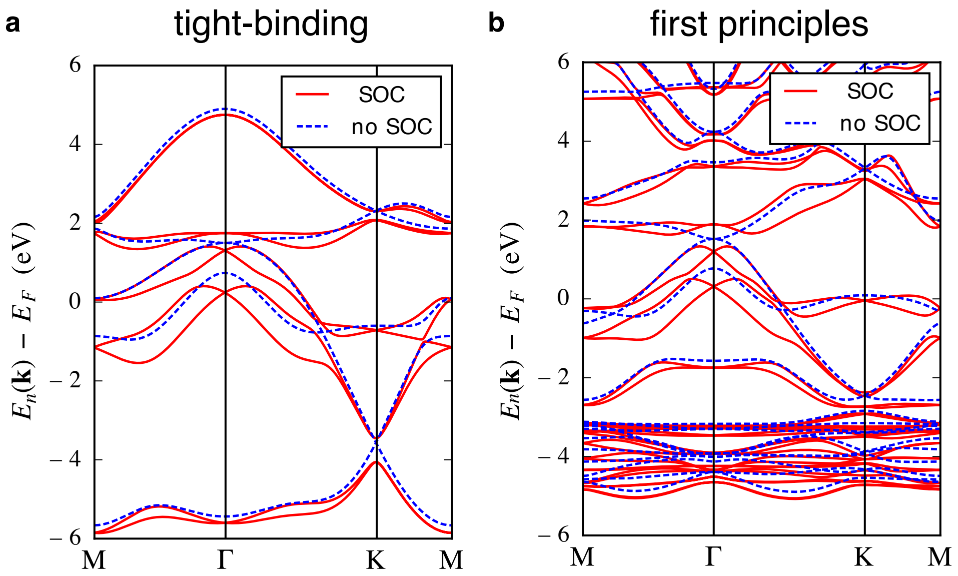

FIG. S1. **Electronic band structures of $\mathbf{BiAg_2}$.** The tight-binding **(a)** and the first-principles **(b)** band structures were calculated with and without taking into account spin-orbit coupling (SOC). The tight-binding parameters were chosen such that the first-principles band structure is closely reproduced (see Supplementary Note 1).

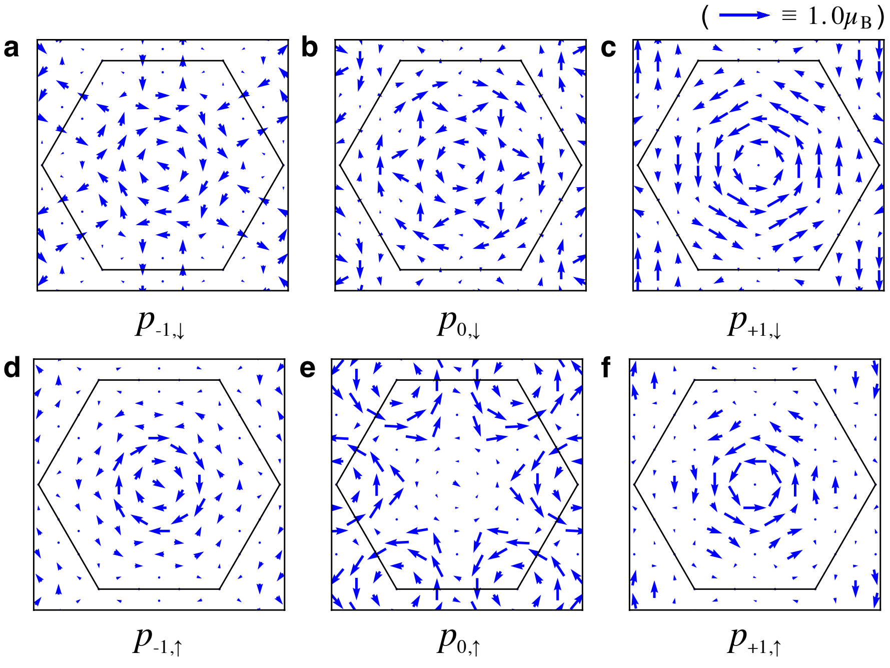

FIG. S2. **Orbital moment of p-derived bands in BiAg$_2$ in the presence of spin-orbit coupling (SOC).** States of distinct orbital symmetry are denoted as p_{-1}, p_0, and p_{+1} following the convention of the main text. Down and up arrows represents lower and upper spin-split bands, respectively. These are degenerate in the absence of SOC. By comparing to the case without SOC shown in Fig. 3 of the main text, we find that overall orbital chirality is unchanged near the Γ-point.

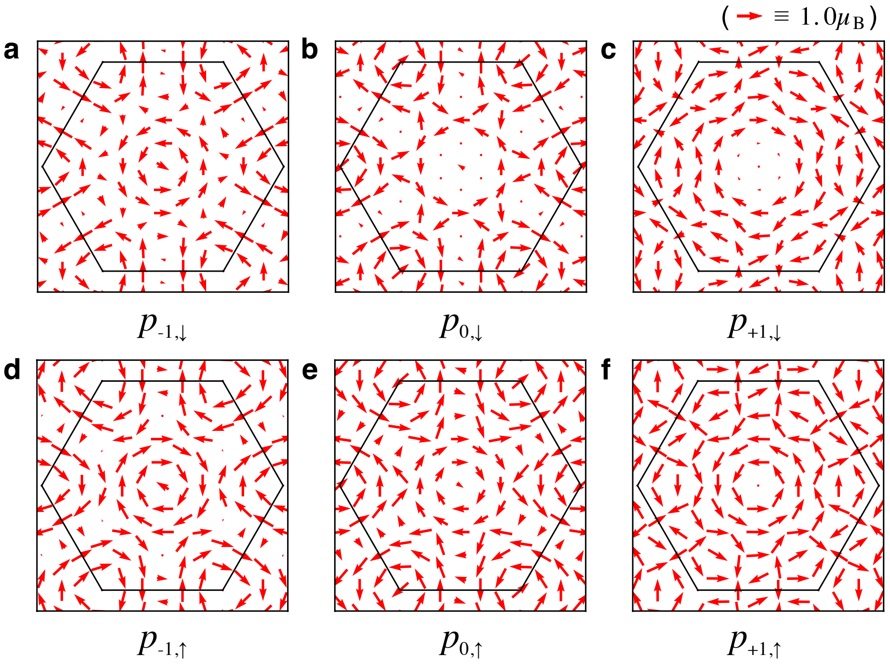

FIG. S3. **Spin moment of p-derived bands in BiAg$_2$ in the presence of spin-orbit coupling (SOC).** Comparing the spin texture of the p-derived bands to the corresponding orbital texture in Fig. S2, we observe that the spin moment is aligned along the opposite(same) direction as the orbital moment of the lower(upper) spin-split bands. Exceptions are the states $p_{+1,\downarrow}$ and $p_{0,\uparrow}$ [(**c**) and (**e**)] since their orbital character is inverted by SOC.

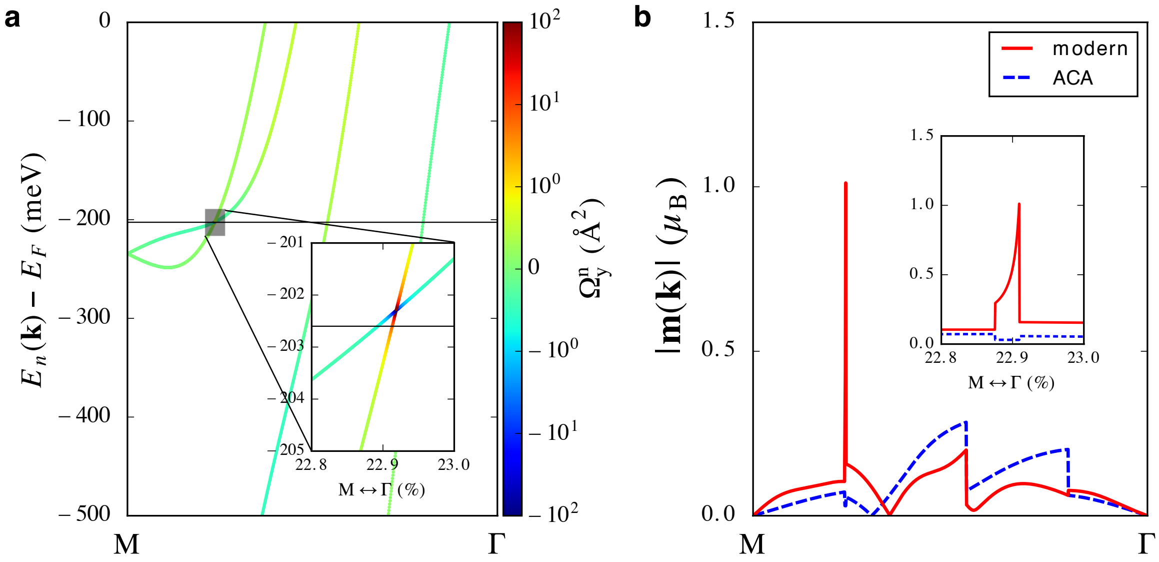

FIG. S4. **Projected Berry curvature and orbital moment (OM) along the MΓ-line.** **(a)** Band structure along the MΓ-line. Colors on top of the first-principles electronic band structure indicate the value of the projected Berry curvature $\langle \partial_{k_y} u_{n\mathbf{k}} | \, z \, | u_{n\mathbf{k}} \rangle$ along the high-symmetry line MΓ. As expected, the projected Berry curvature is pronounced in the vicinity of the band crossing. **(b)** The magnitude of the OM calculated from the modern theory and atom-centered approximation (ACA). The modern theory gives a spiky distribution near the crossing point. Remarkably, both modern theory and ACA results show qualitatively similar behavior apart from the crossing point.

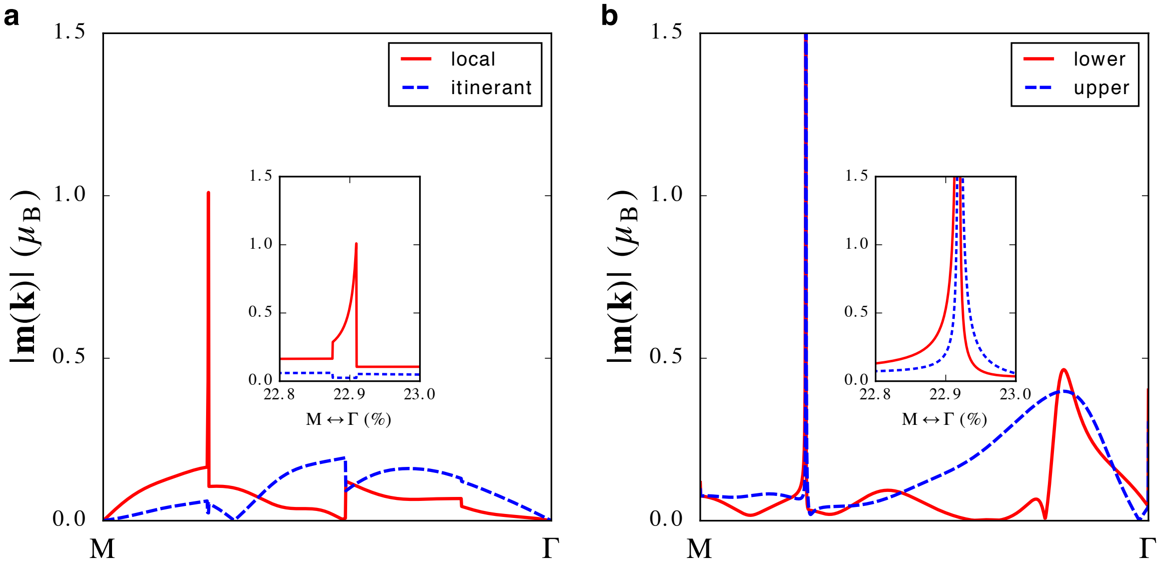

FIG. S5. **Decomposition of the orbital moment (OM) obtained from the modern theory.** **(a)** Decomposition of OM into local and itinerant contributions. The singular behavior of the OM near the band crossing is purely due to the local contribution. **(b)** The individual OM contributions of the upper and lower bands at the crossing. Both lower and upper bands show pronounced values of OM at the crossing point, which confirms that the spiky distribution of the OM is coming from the hybridization between the two crossing bands.
